# Predictors and Mechanisms of Resilience for High School Students with ADHD: A Prospective Longitudinal Study

**DOI:** 10.1007/s10578-024-01704-3

**Published:** 2024-05-15

**Authors:** Elizabeth S. M. Chan, Melissa R. Dvorsky, Cathrin D. Green, Rosanna Breaux, Stephen P. Becker, Joshua M. Langberg

**Affiliations:** 1 Graduate School of Applied and Professional Psychology, Rutgers University, Piscataway, NJ USA; 2 Division of Psychology and Behavioral Health, Children’s National Hospital, Washington, DC USA; 3 Department of Psychiatry and Behavioral Sciences, and Department of Pediatrics, The George Washington University School of Medicine and Health Sciences, Washington, DC USA; 4 Division of Behavioral Medicine and Clinical Psychology, Cincinnati Children’s Hospital Medical Center, Cincinnati, OH USA; 5 Department of Psychology, Virginia Polytechnic Institute and State University, Blacksburg, VA USA; 6 Department of Pediatrics, University of Cincinnati College of Medicine, Cincinnati, OH USA

**Keywords:** Attention-deficit/hyperactivity disorder, Resilience, Adolescence, Self-efficacy, Stress-is-enhancing mindset

## Abstract

Attention-deficit/hyperactivity disorder (ADHD) has primarily been studied from a deficit-focused perspective. However, there are individuals with ADHD who exhibit resilience or a pattern of positive adaptation despite the risks associated with their diagnosis. The present study evaluated whether peer acceptance predicted resilience for adolescents with ADHD and if self-efficacy or a stress-is-enhancing mindset served as mechanisms of those relations. Participants included 113 comprehensively evaluated adolescents with ADHD (67% male) across three time-points (10th–12th grade). Mediation analyses revealed higher T1 peer acceptance significantly predicted higher resilience (β = 0.24) 1.5–2 years later, with higher T2 self-efficacy (β = 0.08) demonstrating a significant indirect effect of the association. A stress-is-enhancing mindset directly predicted resilience (β = 0.15) but was not associated with peer acceptance nor mediated the association between peer acceptance and resilience. Present results are the first to provide longitudinal evidence for peer acceptance, self-efficacy, and a stress-is-enhancing mindset as important for promoting resilience among adolescents with ADHD.

Attention-deficit/hyperactivity disorder (ADHD) has largely been studied from a deficit-focused perspective [20]. Indeed, ADHD symptoms predict a range of peer, family, and academic impairments into adulthood [58], exacting annual illness-related costs of over $100 billion in the United States alone [83]. Moreover, the social and academic impairments associated with ADHD are frequently exacerbated by the increasing demands, socioemotional pressures, and vulnerability to mental health problems associated with adolescence [38]. Importantly, despite the adverse outcomes associated with ADHD, some adolescents with ADHD perform as well as or better than their non-ADHD peers in one or more functional domain(s) [12]. These individuals appear to exhibit *resilience*, or a pattern of positive adaptation, with some thriving despite the neurobehavioral risks associated with their diagnosis [18]. Given this heterogeneity in outcomes, studying ADHD from a *developmental psychopathology* framework that emphasizes both risk and resilience may elucidate strength-based mechanisms that simultaneously promote well-being and positive development while mitigating risk for this population [28, 38].

## Resilience, ADHD, and Peer Acceptance in High School

In the past two decades, the field of resilience has increasingly shifted away from trait-oriented approaches, which view resilience as intrinsic and stable (e.g., personality traits that enhance adaptation to risk; [13]) towards outcome-oriented approaches that view resilience as malleable [53]. Outcome-oriented conceptualizations of resilience additionally require an experience of adversity (e.g., a clinical diagnosis) and positive adaptation despite exposure to that risk [53]. While resilience has been studied extensively in the developmental literature, few studies have examined resilience in ADHD [28]. Nonetheless, within the emerging ADHD and resilience literature, increasing evidence supports peer acceptance (e.g., peer support, close friendships) as protective for this population [28].

For example, in a rigorous longitudinal, multi-informant study of middle school students with ADHD, Dvorsky et al. [28] found peer acceptance moderated the effects of ADHD-related inattention to predict higher grades 18 months later. These findings were robust to baseline grades and cognitive abilities, providing strong evidence for peer acceptance as a protective factor for ADHD-related academic difficulties in middle school. In addition to promoting positive outcomes, longitudinal studies have found social acceptance also mitigates ADHD-related risks, including lower levels of inattention symptoms, irritability, depression, aggression/conduct, and parent- and teacher-rated social problems for elementary and middle school children with ADHD [5, 8, 57]. Cross-sectional findings also suggest that self-reported peer acceptance is associated with higher levels of academic and social competence, lower levels of ADHD symptom severity, and higher levels of self-worth for children at-risk or diagnosed with ADHD [25, 43, 54]. While the construct of peer acceptance can be measured in different ways (e.g., sociometric ratings, assessment of social calendar or size of social network [60]), the aforementioned studies predominantly utilized adolescent self-report as their primary measure of peer acceptance (cf. [8]. Collectively, these studies indicate perceived peer acceptance protects against ADHD-related risks and promotes positive development for elementary and middle school age youth.

In addition, more recently, Chan et al. [17] identified peer acceptance as a top predictor of broad-based positive functioning (i.e., resilience) among a shortlist of individual, family, and social-community positive factors. Although the study had several strengths including multi-informant, multi-method measures as well as a carefully evaluated ADHD group, it was cross-sectional in design. Thus, direction of causality could not be determined (i.e., whether peer acceptance engenders higher levels of resilience or whether more resilient children experience greater peer acceptance in context of ADHD). Moreover, similar to prior research examining the protective effects of peer acceptance in ADHD, the study focused on children, i.e., ages 8–13 years.

Taken together, the relationship between peer acceptance and resilient functioning in older adolescents with ADHD, such as those in high school, remains unknown. This is a critical gap in the literature, as the transition from middle to high school marks a developmental period of increasingly complex and difficult peer relations for students regardless of ADHD status [9]. Moreover, high school students with ADHD are at an increased risk for peer interpersonal difficulties [4], as well as a broad range of adverse outcomes from substance abuse and delinquency to familial disputes to high school dropout [26, 32, 46]. In addition, peer influence may serve as a risk-factor in adolescence, such that adolescents with and without ADHD are more likely to engage in risk-taking behaviors when a peer is present [24, 64]. Given the heightened risk for ADHD-related adverse outcomes, the protective effects of peer acceptance may be insufficient during later adolescence. Indeed, prior ADHD research suggests that the protective effects of a particular mechanism may differ based on developmental stage [19, 59]. Therefore, it is important to examine whether the protective effects of peer acceptance in early adolescence extend into late adolescence, and to study this association longitudinally. Furthermore, youth with ADHD who are more symptomatic often experience higher levels of peer difficulties [1]. Therefore, it is important to examine the potential moderating effect of ADHD symptom severity on the relation between peer acceptance and resilience.

## Self-Efficacy and Stress-is-Enhancing Mindset

Longitudinal investigations additionally enable examination of mechanisms of change through which peer acceptance may promote resilience [28]. To that end, *general self-efficacy* is a particularly promising mechanism through which peer acceptance may contribute to resilience in adolescents with ADHD. General self-efficacy is defined by an individual’s belief in their ability to overcome and achieve goals in their everyday life [75]. It is associated with more adaptive coping strategies and higher levels of well-being and life satisfaction [15, 70]. Prior studies have also found significant associations between self-efficacy and resilience in early to late adolescence (e.g., [40, 42, 65, 69]). Closely related to self-efficacy, Dvorsky [28] found that adolescents with positive self-worth throughout middle and early high school were more likely to have positive social, emotional, and academic outcomes at age fifteen.

Yet due to the myriad of interpersonal, family, and educational difficulties associated with ADHD [58] and negative feedback concerning their abilities [67], ADHD symptoms may moderate the effect of self-efficacy to resilience. Adolescents with ADHD may feel unable to manage their struggles and attempt to apply inadequate coping strategies (e.g., reducing effort, self-isolation). Indeed, studies have found higher levels of ADHD symptoms to be associated with lower levels of self-efficacy [34, 61]. Interpersonal theoretical models (e.g., [82]) also emphasize how the role of negative appraisals from others (i.e., peer rejection) combines with the impact of failure experiences which can cascade into negative self-efficacy particularly for youth with ADHD [30]. In contrast, adolescents with ADHD who experience more peer acceptance may receive more positive feedback that may build greater beliefs about competence and subsequent resilience [51, 71, 84]. Nonetheless, though the prior reviewed evidence suggests that self-efficacy and variables related to peer acceptance (i.e., social support, belonging) collectively predict resilience, to our knowledge there has yet to be an ADHD study examining self-efficacy as a mediator between peer acceptance and resilience.

*Stress mindsets* or differing beliefs about stress may also explain why only some adolescents with ADHD who experience risks associated with their diagnosis exhibit resilience [22]. Specifically, stress mindsets occur on a continuum, and some adolescents with ADHD may hold more of a *stress-is-enhancing mindset* and embrace stressful situations or adversities as opportunities for learning and development [22, 62].^1^ Other adolescents may hold more of a *stress-is-debilitating mindset,* where they experience stress as an impediment likely to lead to negative outcomes [22, 62]. Among adolescents, higher levels of a stress-is-enhancing mindset are associated with greater well-being and adaptive coping strategies [16, 44], and protect against experienced distress and impulsive behaviors frequently associated with increasing adverse experiences [62].

The benefits of this mindset may best be conceptualized from the stress-buffering model [48]. This model posits that, when faced with adversity, individuals with greater support (e.g., peer acceptance) are more likely to bounce-back from the stressor. The social support presumably bolsters cognitive appraisals (e.g., stress-is-enhancing mindset) that increase an individual’s belief that they can cope effectively with the adverse event. For example, the support engendered by peer acceptance may help adolescents rethink stress as potentially beneficial versus detrimental. In turn, a stress-is-enhancing mindset may promote higher levels of resilience to adverse experiences associated with a diagnosis of ADHD. Moreover, adolescents with ADHD who are more symptomatic may experience more stressors and thus require higher levels of a stress-is-enhancing mindset to promote resilience. Nonetheless, to our knowledge there is yet to be a study of stress-is-enhancing mindsets in the context of ADHD.

### Current Study

Taken together, perceived peer acceptance is associated with resilience (in childhood and early adolescence), but whether that effect remains for high school-aged students with ADHD has yet to be studied. Moreover, although preliminary evidence suggests a correlation between peer acceptance and resilience, no study to date has examined this relation longitudinally. In this context, the present study extends prior work by investigating whether peer acceptance longitudinally predicts resilience for high school students with ADHD, and potential mechanisms (self-efficacy, stress-is-enhancing mindset) of those relations. We hypothesized that higher levels of peer acceptance would longitudinally predict higher levels of resilience. We hypothesized that higher levels of self-efficacy and a stress-is-enhancing mindset would mediate that relation. Exploratory analyses were additionally conducted to examine whether the relation between peer acceptance, self-efficacy, and a stress-is-enhancing mindset to resilience is moderated by ADHD symptom severity.

## Methods

### Participants and Procedures

Participants were 113 adolescents (67% male) from two sites in the Southeastern and Midwestern United States. Adolescents and their parents were recruited across two cohorts collected from two consecutive years (2016 and 2017) when students were in 8th grade and followed until the end of 10th grade for a prospective longitudinal study examining sleep in adolescents with ADHD [5, 6, 49]. Participants from both cohorts who consented to be contacted were then invited to participate in a follow-up longitudinal study utilizing online surveys (90.8% retention rate), following adolescents into 11th or 12th grade. Three time points were used in the present study: fall/winter 10th grade for our primary predictor (i.e., peer acceptance) and demographic covariates (T1; *M*_age_ = 15.71, *SD* = 0.39), spring of 10th or 11th grade for our mediators self-efficacy and stress-is-enhancing mindset (T2; *M*_age_ = 16.77, *SD* = 0.59), and spring of 11th or 12th grade for follow-up (T3; *M*_age_ = 17.53, *SD* = 0.58). Participant characteristics for the current sample include 81.3% White/Non-Hispanic, 8.0% Black, 8.0% Biracial/Multiracial, 1.8% Asian, and 0.9% American Indian/Alaskan Native. Participant income ranged from $5,000–125,000 (*M* = $86,858, *SD* = $35,268).

The study inclusion criteria included estimated Full Scale IQ ≥ 80 as assessed by the Wechsler Abbreviated Scale of Intelligence, Second Edition [78] and meeting full Diagnostic and Statistical Manual for Mental Disorders, Fifth Edition [2] criteria for either ADHD combined or predominantly inattentive presentation. Exclusion criteria were a previous diagnosis or meeting criteria for autism spectrum disorder, bipolar disorder, or a dissociative or psychotic disorder; or previous diagnosis of an organic sleep disorder (e.g., obstructive sleep apnea). During the initial assessment, all participants underwent a comprehensive ADHD diagnostic evaluation including administration of the Children’s Interview for Psychiatric Syndromes [79] to the parent and adolescent and the Vanderbilt ADHD rating scale was completed by teachers and parents. Adolescents and parents provided consent and assent for participation and were compensated for participation at all timepoints. This study was approved by the Cincinnati Children’s Hospital Medical Center and Virginia Commonwealth University Institutional Review Boards.

### Measures

*ADHD Symptoms.* Parents completed the Vanderbilt ADHD Diagnostic Rating Scale (VADRS) at T1 to assess for DSM-5 based symptoms of inattention and hyperactivity/impulsivity [80], with total ADHD symptoms used in the present study. The VADPRS has good internal consistency, factor structure, and concurrent validity for the assessment of ADHD [80]. Symptoms are rated on a 4-point scale ranging from 0 = *Never* to 3 = *Very Often*, with symptoms rated as occurring often or very often counting as being clinically present. ADHD total symptoms were used in the current study and reliability was good (α = 0.92).

*Peer acceptance*. Adolescents completed the Self-Perception Profile for Adolescents (SPPA) [35] at T1, with the social acceptance domain used in the present study. The SPPA is a well-validated measure, with good validity and reliability, and concurrent validity with other measures of social functioning [36]. The SPPA uses a “some kids/other kids” format on a 4-point Likert scale, with higher scores indicating greater perceived peer acceptance. Items focused on success in the peer domain (e.g., ability to make/maintain friends, popularity with peers). In the present study, mean scores on social acceptance scale were used (α = 0.72).

*Self-Efficacy*. Adolescents completed the General Self-Efficacy Scale (GSE; 74) at T2. The GSE is comprised of 10-items and assesses beliefs regarding ability to respond to new or difficult situations (e.g., “I can solve most problems if I invest the necessary effort”). Ratings are made on a 4-point Likert scale (1 = *Not at all true* to 4 = *Exactly true*) with higher scores indicating greater self-efficacy. The scale has strong psychometric properties and is widely used with adolescents [73, 50], and current study α = 0.91.

*Stress-is-enhancing mindset*. Adolescents completed the Stress Mindset Measure Short (SMM-S) at T2 [22]. The SMM-S is a 3-item measure that assesses personal beliefs on whether stress leads to learning and growth, health and energy, and productivity (e.g., experiencing stress improves your learning and growth; experiencing stress improves your productivity; experiencing stress improves your health/energy) and were selected to be appropriate for adolescent samples [62]. Ratings are made on a 6-point Likert scale from 0 = *Strongly Disagree* to 5 = *Strongly Agree*, with higher scores indicating greater stress-is-enhancing mindset. The SMM-S demonstrates good reliability [62] and the present observed alpha was 0.87.

*Resilience*. Adolescents completed the Brief Resilience Scale [77] at T2 and T3. The scale has demonstrated good alpha reliability in prior studies and excellent convergent and discriminant validity [47, 55, 77]. Adolescents responded to 6-items (e.g., “I tend to bounce back quickly after hard times”; “It does not take me long to recover from a stressful event”; “I usually come through difficult times with little trouble”) on a 5-point Likert scale (1 = *Strongly Disagree* to 5 = *Strongly Agree*), with higher scores indicating greater resilience. Mean scores were used in the current study (α = 0.85).

*Demographic variables.* Parents reported on family income as well as adolescent sex (male = 0; female = 1), race, and ethnicity in a demographic form.

### Analytical Approach

Bivariate correlation analyses were conducted to examine whether any baseline (T1) participant demographic characteristics (i.e., sex, income, IQ, and age) were significantly associated with the follow-up (T3) resilience outcome. Variables correlated with our resilience outcome variable at *p* < 0.05 were retained for all subsequent analyses.

To investigate our primary study aim, mediation analyses guided by the modeling strategies described by Hayes and colleagues [37] were used. Specifically, the MEDIATE macro for SPSS [37] was used to test whether T1 peer acceptance predicts T3 resilience, and if that relation is mediated by T2 stress-is-enhancing mindset or self-efficacy. Separate models were run for each mediator (self-efficacy, stress-is-enhancing mindset) with 10,000 bootstrapped samples [66]. ADHD symptom severity, cohort, significant covariates, and baseline resilience collected at T2 were included in each mediation model. For these analyses, the indirect effects are considered significant if the 95% CIs did not encapsulate zero.

For our exploratory aim, hierarchical regression and moderation analyses was used to examine the moderating effect of ADHD symptom severity to our primary constructs of interest (peer acceptance, self-efficacy, and stress-is-enhancing mindset) to resilience. Separate models were run for each predictor. Specifically, hierarchical regressions were conducted to determine whether a significant interaction between ADHD symptom severity and peer acceptance, self-efficacy, or stress-is-enhancing mindset, respectively, was present. To probe a significant interaction effect, the PROCESS macro for SPSS (2021) was used. The macro estimates coefficients and standard error for the outcome from the predictor, moderator, and interaction of the two with control variables. Simple slopes for the effects of the predictor on the outcome at specified values (1 standard deviation above and below the mean) of the moderator were then calculated. A visual plot of the interaction was then produced by imputing results into a graphical interface.

## Results

### Preliminary Analyses

All independent/dependent variables were screened for univariate outliers, defined as values greater than 3 *SD* above/below the within-group mean. Six (0.46%) datapoints were identified as outliers and corrected to the most extreme value 3 *SD* above/below the within-group mean. Four-point seven percent of data were missing completely at random (Little’s MCAR test: *χ2* = 66.59, *p* = 0.07). Missing data was imputed using expectation maximization based on all available data. Bivariate correlations revealed sex was significantly correlated with T3 resilience with females reporting significantly lower resilience than males (*p* < 0.001), and therefore sex was included in all subsequent models. No other adolescent characteristics were significantly associated with the outcome (*p*s > 0.22; see correlation matrix in Table 1).

**Table 1:** Means, standard deviations, and intercorrelations among study variables

	1	2	3	4	5	6	7	8	9	10	11
1. T3 resilience	–	− 0.04	− 0.12	− 0.11	.04	− 0.30***	− 0.13	0.37***	0.48**	0.52***	0.27**
2. Cohort	− 0.04	–	− 0.02	− 0.02	− 0.01	− 0.10	0.10	0.10	0.02	− 0.06	0.04
3. Age	− 0.12	− 0.02	–	− 0.12	0.06	0.01	− 0.03	0.04	0.06	0.02	− 0.01
4. IQ	− 0.11	− 0.02	− 0.12	–	0.32***	0.008	− 0.03	− 0.10	0.09	0.03	− 0.10
5. Income	0.04	− 0.01	0.06	0.32***	–	0.001	− 0.25**	0.11	0.14	0.24*	− 0.02
6. Sex	− 0.30***	− 0.10	0.01	0.008	0.001	–	− 0.04	− 0.06	− 0.28**	− 0.11	− 0.19*
7. ADHD	− 0.13	0.10	− 0.03	− 0.03	− 0.25**	− 0.04	–	− 0.07	− 0.06	− 0.09	0.21*
8. T1 peer acceptance	0.37***	0.10	0.04	− 0.10	0.11	− 0.06	− 0.07	–	0.27**	0.34**	− 0.05
9. T2 resilience	0.48***	0.02	0.06	0.09	0.14	− 0.28**	− 0.06	0.27**	–	0.43***	− 0.07
10. T2 self-efficacy	0.52***	− 0.06	0.02	0.03	0.24*	− 0.11	− 0.09	0.34***	0.43***	–	0.06
11. T2 stress-is-enhancing mindset	0.27**	0.04	− 0.01	− 0.10	− 0.02	− 0.19*	0.21*	− 0.05	− 0.07	0.06	–
M	3.42	–	15.71	105.72	86858.41	–	4.30	2.80	3.41	2.80	1.50
SD	0.64	–	0.39	13.08	35268.07	–	4.06	0.71	0.75	0.55	1.31

Demographic variables were collected at baseline (T1). T1 is fall of 10th grade, T2 is spring of 10th/11th grade, and T3 is spring of 11th/12th grade. ADHD = total ADHD symptoms.  Sex: 0 = male and 1 = female

*p < 0.05, **p < 0.01, ***p < 0.001

### Mediation Analyses

**Fig. 1:**
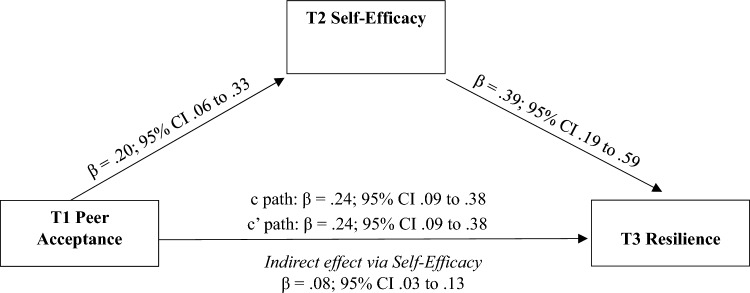
Indirect effect of T1 peer acceptance to T3 resilience via T2 self-efficacy. Analyses controlled for adolescent sex, ADHD symptoms, baseline resilience, and cohort. The c path coefficient represents the total effect of peer acceptance and self-efficacy on resilience. The c-prime path coefficient refers to the direct effect of peer acceptance on resilience. Effects are significant if their 95% CIs do not contain zero (solid line)

*Self-efficacy:* Results of the bias-corrected, bootstrapped conditional effects models revealed significant direct effects from higher levels of peer acceptance (c’ path: β = 0.24; 95% CI 0.09–0.38**)** and self-efficacy (β = 0.39; 95% CI 0.19–0.59) to greater resilience when covarying cohort, sex, ADHD symptoms, and baseline resilience (Fig. 1). There was a significant total effect (c path: β = 0.24; 95% CI 0.09–0.38) and a significant indirect effect (β = 0.08; 95% CI 0.03–0.13) from peer acceptance and resilience via self-efficacy; thus, indicating mediation was present.

**Fig. 2:**
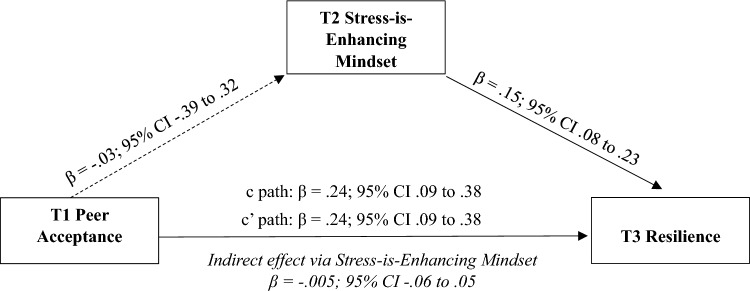
Indirect effect of T1 peer acceptance to T3 resilience via T2 stress-is-enhancing mindset. Analyses controlled for adolescent sex, ADHD symptoms, baseline resilience, and cohort. The c path coefficient represents the total effect of peer acceptance and stress-is-enhancing mindset on resilience. The c-prime path coefficient refers to the direct effect of peer acceptance on resilience. Effects are significant if their 95% CIs do not contain zero (solid line)

*Stress-is-enhancing mindset.* Results of the bias-corrected, bootstrapped model covarying cohort, sex, ADHD symptoms, baseline resilience, revealed significant direct effects from peer acceptance (β = 0.24; 95% CI 0.09–0.38) and stress-is-enhancing mindset (β = 0.15; 95% CI 0.08–0.23) to resilience (Fig. 2). In addition, the total effect (c path: β = 0.24; 95% CI 0.09–0.38), but not the indirect effect (β = − 0.005; 95% CI − 0.06 to 0.05), from peer acceptance and resilience via stress-is-enhancing mindset was significant; thus, indicating mediation was not present.

### Hierarchical Regression and Moderation Analyses

Results of all regression models are displayed in Table 2.

**Table 2:** Buffering effects of peer acceptance, stress-is-enhancing mindset, and self-efficacy to resilience at follow-up

	DV: T3 Resilience						DV: T3 Resilience				
	*Total R*^*2*^	*R*^*2*^_*inc*_	*B*	*SE*	*β*		*Total R*^*2*^	*R*^*2*^_*inc*_	*B*	*SE*	*β*
*Step 1:*						*Step 1:*					
Cohort			− 0.08	.11	− 0.07	Cohort			− 0.08	0.12	− 0.07
Sex			− 0.25	.17	− 0.19*	Sex			− 0.25	0.12	− 0.19*
T2 Resilience			0.36	0.07	.43***	T2 Resilience			.36	0.07	.43***
*Step 2:*	0.27	0.27				*Step 2:*	0.27	0.27			
T1 ADHD			− 0.02	0.01	− 0.11	T1 ADHD			− 0.02	0.01	− 0.11
*Step 3:*	0.34	0.07**				*Step 3:*	0.39	0.12***			
T1 Peer Acceptance (PA)			0.24	0.07	0.26**	T2 Self-Efficacy (SE)			0.45	0.10	0.39***
*Step 4:*	0.36	0.02*				*Step 4:*	0.40	0.01			
PA x ADHD			− 0.03	.02	− 0.15*	SE x ADHD			0.03	0.03	0.08

	DV: T3 Resilience				
	*Total R*^*2*^	*R*^*2*^_*inc*_	*B*	*SE*	*β*
*Step 1:*					
Cohort			− 0.08	.11	− 0.07
Sex			− 0.25	.17	− 0.19*
T2 Resilience			0.36	0.07	0.43***
*Step 2:*	0.27	0.27			
T1 ADHD symptoms			− 0.02	.01	− 0.11
*Step 3:*	0.36	0.09***			
T2 Stress-is-enhancing mindset (SEM)			0.15	0.04	0.31***
*Step 4:*	0.40	0.04*			
SEM × ADHD			0.02	0.008	0.22*

Variables in prior steps are included in the model at the next step but are not presented in the table for space. *R*^*2*^ change of .01 = small, .06 = medium, and .14 = large [21]

*p < 0.05, **p < 0.01, ***p < 0.001

*Peer acceptance.* Consistent with the above results, higher levels of peer acceptance (Step 3) explained an incremental 24% increase in resilience (*p* = 0.003), over and above sex, cohort, and baseline resilience (Step 1) and total ADHD symptoms (Step 2). The interaction between peer acceptance with ADHD symptom severity was also significant (*β* = -0.15, *p* = 0.05; Step 4). Probing the interaction revealed ADHD symptoms did not moderate the positive relation between greater peer acceptance and resilience (p = 0.17), though individuals with fewer ADHD symptoms were more likely to experience high (+ 1 SD) levels of peer acceptance (*p* < 0.001).

*Self-Efficacy.* Higher levels of self-efficacy (Step 3) explained an incremental 39% increase in resilience at follow-up (*p* < 0.001), above and beyond sex, cohort, and baseline resilience and total ADHD symptoms (Steps 1–2). There was no significant interaction between self-efficacy and ADHD symptoms (*β* = 0.08, *p* = 0.29; Step 4).

**Fig. 3:**
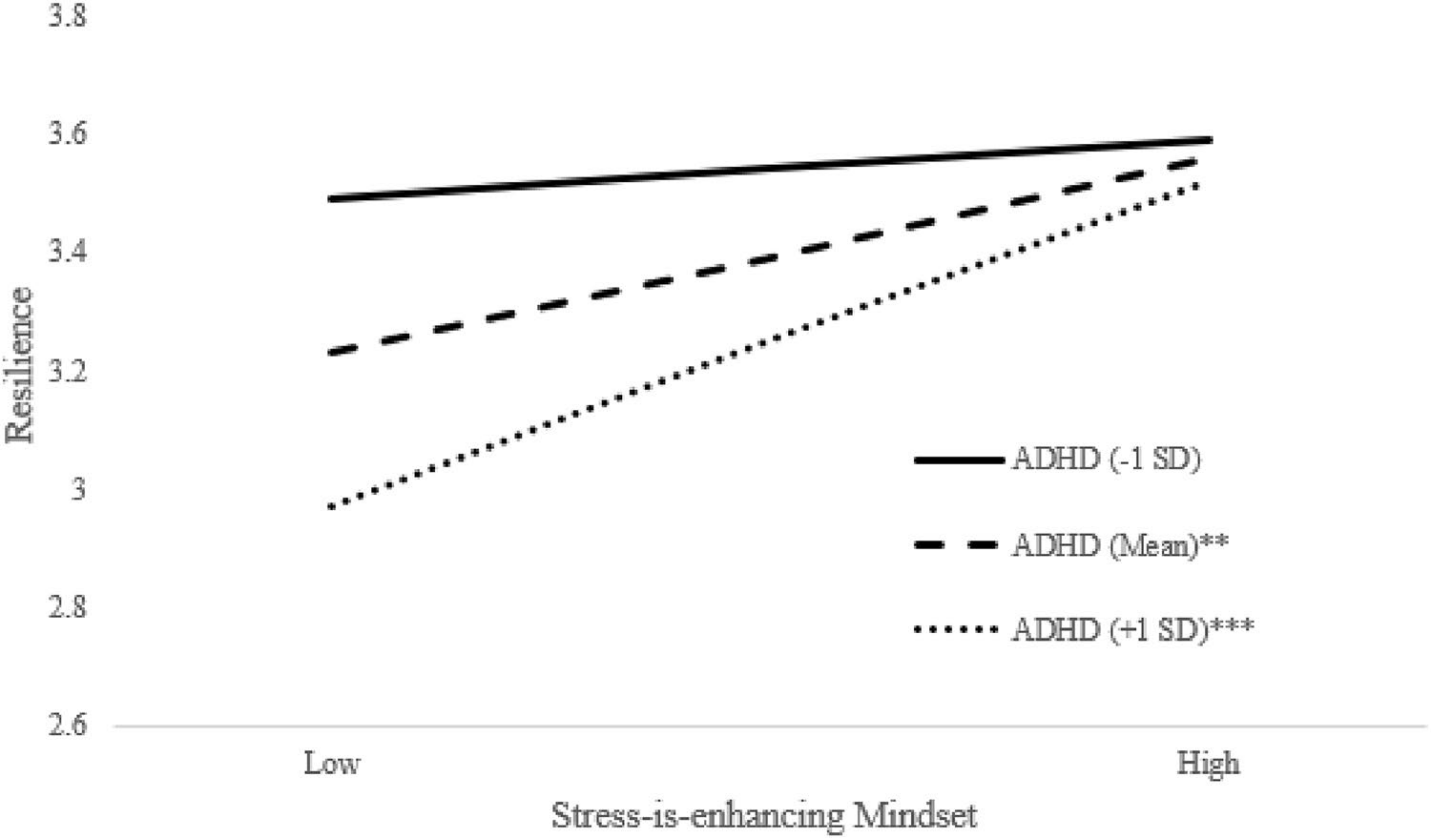
ADHD symptom severity moderates the longitudinal association between stress-is-enhancing mindset to resilience. One SD below or above the mean were used to characterize Low and High stress-is-enhancing mindset and Low and High total ADHD symptoms. Analyses controlled for adolescent sex, baseline resilience, and cohort. **p <.01, ***p < .001

*Stress-is-enhancing mindset.* A greater stress-is-enhancing mindset (Step 3) explained an incremental 31% increase in resilience (*p* < 0.001), over and above sex, cohort, and baseline resilience (Step 1) and total ADHD symptoms (Step 2). The interaction between stress-is-enhancing mindset with ADHD symptom severity was also significant (*β* = 0.22, *p* = 0.01; Step 4). A visual plot of the interaction (see Fig. 3) revealed that for adolescents with moderate (*mean*) and high (+ *1 SD*) ADHD symptoms, at least a moderate or high stress-is-enhancing mindset was needed to promote resilience (all *p* < 0.002).

## Discussion

This study is the first prospective longitudinal evaluation of peer acceptance and resilience and hypothesized mechanisms (self-efficacy, stress-is-enhancing mindset) in a comprehensively diagnosed sample of high school students with ADHD. Study results revealed peer acceptance in 10th grade predicted almost a quarter (24%) of the variance in resilience 1.5–2 years later over and above baseline resilience and ADHD symptom severity. Our finding is aligned with prior ADHD research establishing peer acceptance as a predictor of resilient trajectories for elementary and middle school students [17, 25, 29, 54]. Current results contribute to the ADHD literature by demonstrating the predictive effects of peer acceptance extend upwards into late adolescence, despite the social impairments frequently associated with ADHD, and the increasingly complex peer relationships for this age group [56]. Taken together, these findings point to peer acceptance as a critical factor for promoting resilience among older adolescents with ADHD.

Results further revealed self-efficacy mediated the above reported positive relation from baseline peer acceptance to follow-up resilience at the end of high school. These findings are consistent with both intervention and cross-sectional findings which indicate support from peers promote self-efficacy for adolescents with neurodevelopmental disabilities, including ADHD [51, 84]. Indeed, adolescents with ADHD who experience peer acceptance may receive more positive feedback which may build beliefs about competence and subsequent resilience [Saarni 1999]. The mediation model additionally revealed self-efficacy directly accounted for 39% of the variance in resilience. This sizeable effect is consistent with evidence from the developmental literature linking self-efficacy and resilience [40, 42]. Similarly, greater self-efficacy has been associated with positive outcomes for children and adolescents with ADHD, including lower levels of depression and internalizing symptoms (McQuade et al., 2011 [59], and reported higher quality of life [72]. Our findings on the role of self-efficacy are also consistent with findings from prior work demonstrating the importance of self-worth in predicting subsequent adaptive outcomes in adolescents with ADHD [30].

Finally, although a stress-is-enhancing mindset directly predicted resilience, it was not associated with peer acceptance nor a mediator for peer acceptance and resilience. The latter findings are aligned with developmental research demonstrating that cognitive appraisals or mindsets related to success and failures may be more influenced by caregivers and teachers than peers [39, 63]. For example, the more parents and teachers praised a student’s effort versus their ability, the more likely an individual held appraisals similar to a stress-is-enhancing mindset (e.g., challenge is associated with growth; [39, 63]). In contrast, preliminary evidence indicates peer-modeled mindsets via more direct methods such as demonstration or advice-giving can shift students’ mindsets towards adversity [41]. Lastly, a small subset of youth with ADHD exhibits a positive bias regarding their abilities [11]. For these youth a stress-is-enhancing mindset may not reflect an objective ability to handle stress in improving their productivity/growth and in turn resilience. It will be important for future research to include other (e.g., parent, teacher, or objective) assessments of stress-is-enhancing mindset. Nonetheless, given the overall strong link between a stress-is-enhancing mindset to resilience in this study, future research may examine whether this mindset can be bolstered through peer modeling, as peer acceptance alone appears insufficient for development of adaptive appraisals of stress.

In addition, the significant, positive effect of a stress-is-enhancing mindset to resilience was moderated by ADHD symptom severity. Specifically, for adolescents with moderate and high ADHD symptoms, at least a moderate or high stress-is-enhancing mindset was needed to promote resilience. This result may speak to the high degree of impairment associated with ADHD for some adolescents [38], which may require a stronger stress-is-enhancing mindset in order to yield resilience advantages for this high-risk population. To our knowledge, this is the first study to examine a stress-is-enhancing mindset in individuals with ADHD. However, our findings are consistent with ADHD studies associating positive outcomes (e.g., greater well-being and academic outcomes; [14, 52]) with a *growth mindset,* defined as the belief that abilities are malleable [31], and akin to the stress-is-enhancing mindset in its growth orientation. Indeed, recent intervention work from the developmental literature indicate ‘synergistic’ interventions targeting both stress-is-enhancing and growth mindsets yield significant effects in decreasing physiological reactions to stress, psychological well-being, and academic success in high school and college students [81]. Additional research is needed to evaluate the impact of a stress-is-enhancing mindset to adaptive outcomes for adolescents with ADHD, and whether extant interventions targeting this mindset (e.g., [23, 45, 81]) are effective for this population.

### Limitations

Along with notable strengths, certain study limitations and areas for future study warrant consideration. A significant study strength is our focus on resilience and identifying positive mechanisms of resilience in the context of ADHD. However, our sample size was moderate and replication of current findings with a larger sample is needed. We also utilized self-report measures for our primary study variables (peer acceptance, self-efficacy, stress-is-enhancing mindset), as literature indicates these constructs are best measured by self-report during adolescence and emerging adulthood [10, 27]. Nonetheless, future research would benefit from examining current findings with multi-informant and/or more objective measures (e.g., observation, sociometric ratings, size of social networks) of the assessed constructs (e.g., resilience, peer acceptance). In addition, social skills impairment in addition to ADHD symptomatology may predict peer acceptance, and future research would benefit from including social impairment as a covariate in analyses. Another significant study strength was our longitudinal design, which enabled us to be better able to infer causality between peer acceptance and resilience for high school students with ADHD, as well as hypothesized mechanisms. However, future research may benefit from applying a developmental cascade framework to determine reciprocal relations between the examined variables (e.g., utilizing longitudinal cross-lagged models to examine whether greater self-efficacy builds greater resilience and vice versa [28]). It is also important for future work to examine within and between effects to capture individual differences in mechanisms of resilience using person-centered analytic approaches. Furthermore, although study variables (peer acceptance, self-efficacy, stress-is-enhancing mindset) predicted significant variance in resilience (15–39%) for adolescents with ADHD, there remains up to 61% of variance that our models did not account for. Consistent with social-ecological models of resilience, a broader array of individual, peer, and community variables need to be examined as predictors of resilience. Similarly, given the limited sociocultural diversity of the present sample, future research examining the present study questions in more diverse samples is needed to increase generalizability of findings, as culture and diversity have been identified as important aspects of resilience in previous literature [7].

### Summary

The current study is the first to establish peer acceptance as a predictor of resilience and self-efficacy as a mechanism to explain that association in high school students with ADHD. To our knowledge, we are also the first to examine the benefits of a stress-is-enhancing mindset in the context of ADHD. Collectively, study results indicate all three examined constructs (peer acceptance, self-efficacy, stress-is-enhancing mindset) are substantial predictors of increased resilience. Taken together, study results suggest peer acceptance, self-efficacy, and a stress-is-enhancing mindset are important novel constructs for promoting resilience and more adaptive outcomes broadly for high school students with ADHD. Future research may benefit from examining interventions that specifically target these mechanisms in context of ADHD. As a starting point, promising interventions for promoting peer acceptance among youth with ADHD, include treatments that target self-regulation, friendship coaching, and social problem solving, which have yielded greater improvements in social functioning than traditional social skills training for students with ADHD [33, 68, 76]. Preliminary evidence also indicates interventions that address social-emotional difficulties may improve aspects of positive self-concept, including self-esteem which is closely related to self-efficacy [3]. Lastly, future research may benefit from evaluating the efficacy of extant interventions within the developmental literature that target a stress-is-enhancing mindset (e.g., [23, 45, 81]) for adolescents with ADHD. Altogether, study findings highlight the importance of targeting not only individual level factors, but also broader, structural factors that may enhance well-being for individuals with ADHD [17, 28].

## Footnotes

1 Both self-efficacy and a stress-is-enhancing mindset predict the use of more adaptive coping strategies in response to adversity [17, 69, 24, 62]. However, self-efficacy relates to an adolescent’s specific belief in their ability to problem solve and be resourceful in response to adversity [71]. In contrast, a stress-is-enhancing mindset relates to interpreting feelings of stress as a learning opportunity, which can, but not necessarily, include developing more adaptive problem-solving strategies [24].
